# Overexpression of the *Drosophila* ATR homologous checkpoint kinase Mei-41 induces a G2/M checkpoint in *Drosophila* imaginal tissue

**DOI:** 10.1186/s41065-018-0066-4

**Published:** 2018-09-06

**Authors:** Fabienne E. Bayer, Mirjam Zimmermann, Anette Preiss, Anja C. Nagel

**Affiliations:** Universität Hohenheim, Institut für Genetik, Garbenstr. 30, 70599 Stuttgart, Germany

**Keywords:** ATR, DNA damage checkpoint, Mei-41, Overexpression, p53 activation

## Abstract

**Background:**

DNA damage generally results in the activation of ATM/ATR kinases and the downstream checkpoint kinases Chk1/Chk2. In *Drosophila melanogaster,* the ATR homologue *meiotic 41* (*mei-41)* is pivotal to DNA damage repair and cell cycle checkpoint signalling. Although various *mei-41* mutant alleles have been analyzed in the past, no gain-of-function allele is yet available. To fill this gap, we have generated transgenic flies allowing temporal and tissue-specific induction of *mei-41.*

**Results:**

Overexpression of *mei-41* in wing and eye anlagen affects proliferation and a G2/M checkpoint even in the absence of genomic stress. Similar consequences were observed following the overexpression of the downstream kinase Grapes (Grp) but not of Loki (Lok), encoding the respective *Drosophila* Chk1 and Chk2 homologues, in agreement with their previously reported activities. Moreover, we show that irradiation induced cell cycle arrest was prolonged in the presence of ectopic *mei-41* expression. Similar to irradiation stress, *mei-41* triggered the occurrence of a slower migrating form of Grp, implying specific phosphorylation of Grp in response to either signal. Using a p53R-GFP biosensor, we further show that overexpression of *mei-41* was sufficient to elicit a robust p53 activation in vivo.

**Conclusion:**

We conclude that overexpression of the *Drosophila* ATR homologue *mei-41* elicits an effectual DNA damage response irrespective of irradiation.

**Electronic supplementary material:**

The online version of this article (10.1186/s41065-018-0066-4) contains supplementary material, which is available to authorized users.

## Background

Environmental and intrinsic stressors may impact the integrity of genome, i.e. the DNA, thereby provoking mutations eventually leading to cellular transformation or cell death. DNA damage is combated by a complex interplay of repair mechanisms ensuring the stability of the genome. Studies on DNA damage response (DDR) in a large variety of organisms, be it single cells like yeast or multicellular organisms like *Drosophila* or mammals, revealed that all organisms have evolved a core of components strikingly conserved across eukaryotes (reviewed in [1–3]). DDR can be envisaged as a cascade of signalling events, starting with the recognition of DNA lesions followed by the activation of the DNA damage checkpoint pathway to cause a temporarily cell cycle arrest thus enabling DNA repair processes to occur (reviewed in [1, 4]). Typical of signalling cascades, DDR is regulated by phosphorylation events mediated by different kinases all belonging to the conserved phosphatidylinositol 3-kinase related protein kinase (PIKK) superfamily (reviewed in [5, 6]). These kinases transmit the signals from the site of DNA damage to the cell cycle machinery by activating cell cycle checkpoints. The G2/M DNA damage checkpoint is critical for the maintenance of genome stability as unrepaired DNA double strand breaks (DSB) may directly cause mistakes in chromosomal segregation to the daughter cells if ending up in the M phase of the cell cycle. Hence, the final exit strategy in multicellular organisms before cellular transformation, and eventually cancer occurs as consequence of DDR is cellular suicide, i.e. apoptosis (reviewed in [7, 8]).

The Ataxia-Telangiectasia Mutated (ATM) and ATM and Rad3-related (ATR) kinases are the central mediators of DDR (reviewed in [5, 6]). ATM is known to orchestrate a global response to DSB in higher organisms including DNA repair, checkpoint activation and apoptosis. Well characterized targets of ATM are the Chk2 kinase and the tumour suppressor p53, the latter being stabilized upon DNA damage to further initiate specific target gene expression executing cell cycle arrest, DNA repair and apoptosis, respectively (reviewed in [1, 9]). Whereas ATM is involved primarily in the mammalian DSB response, ATR is activated by a much wider range of genotoxic stresses and appears to be a much more important player in DDR of yeast cells than ATM (reviewed in [2, 6]). Once activated, ATR phosphorylates and activates the protein kinase Chk1, which effects a cell cycle arrest at the G2/M transition, allowing more time for DNA repair so that cells do not enter mitosis prematurely. Noteworthy, there is considerable crosstalk between the ATM/ATR signal transduction pathways (reviewed in [6, 8]).

The *Drosophila* homologue of ATM is called *telomere fusion* (*tefu)*, as it was originally identified by its essential role in telomere maintenance [10]. Although *tefu* is important for p53 activation and DNA damage-induced apoptosis, it has no evident role in cell cycle arrest in response to DNA damage [10–14]. Similar to vertebrates, the *Drosophila* Chk2 homologue *loki* (*lok*) regulates p53-mediated apoptosis in response to DNA damage as well as to telomere loss [15–21]. The ATR homologue in *Drosophila* is encoded by *meiotic 41* (*mei-41).* Mutational analyses revealed that *mei-41* is indispensable for meiotic recombination checkpoints as well as for DNA damage checkpoints in somatic cells [13, 22–27]. Like its target kinase *grapes* (*grp*) (the *Drosophila* Chk1 homologue), *mei-41* is important to postpone the mitosis entry in larval cells after IR-stress [24, 25, 28]. Moreover, *mei-41* and g*rp* mutant flies are highly sensitive towards triggers that damage DNA or inhibit DNA replication, and are therefore essential to maintain genomic and chromosomal stability [29–33]. Overall in *Drosophila, mei-41* appears to mostly fulfil the roles of both ATM and ATR with regard to DDR, whereas *tefu*’s primary role is the maintenance of telomeres and triggering apoptosis.

Although various *mei-41* mutant alleles have been analyzed in the past in *Drosophila*, no gain-of-function allele is yet available. In order to fill this gap, we generated a *mei-41* construct under UAS-control, which allows temporal and tissue-specific expression of *mei-41* with the help of the versatile Gal4/UAS system [34]. We show that the overexpression of *mei-41* in imaginal tissues is sufficient to induce a G2 arrest constraining the growth of affected tissues. Moreover, in the presence of ectopic Mei-41 cells are hampered to resume the cell cycle after irradiation (IR)-mediated arrest. Upon IR-stress, Grp protein shows retarded mobility, and likewise upon *mei-41* overexpression, suggesting that ectopic Mei-41 protein is sufficient to phosphorylate Grp protein. Finally, using a p53-biosensor we show that overexpression of *mei-41* effects p53 reporter gene expression in vivo*,* suggesting a link to the Chk2/*lok* pathway as well. Overall, our data provide evidence for a *mei-41*-induced cellular response independent of DDR-mediated *mei-41* activation.

## Results

### Generation of a mei-41 overexpression construct

**Fig. 1:**
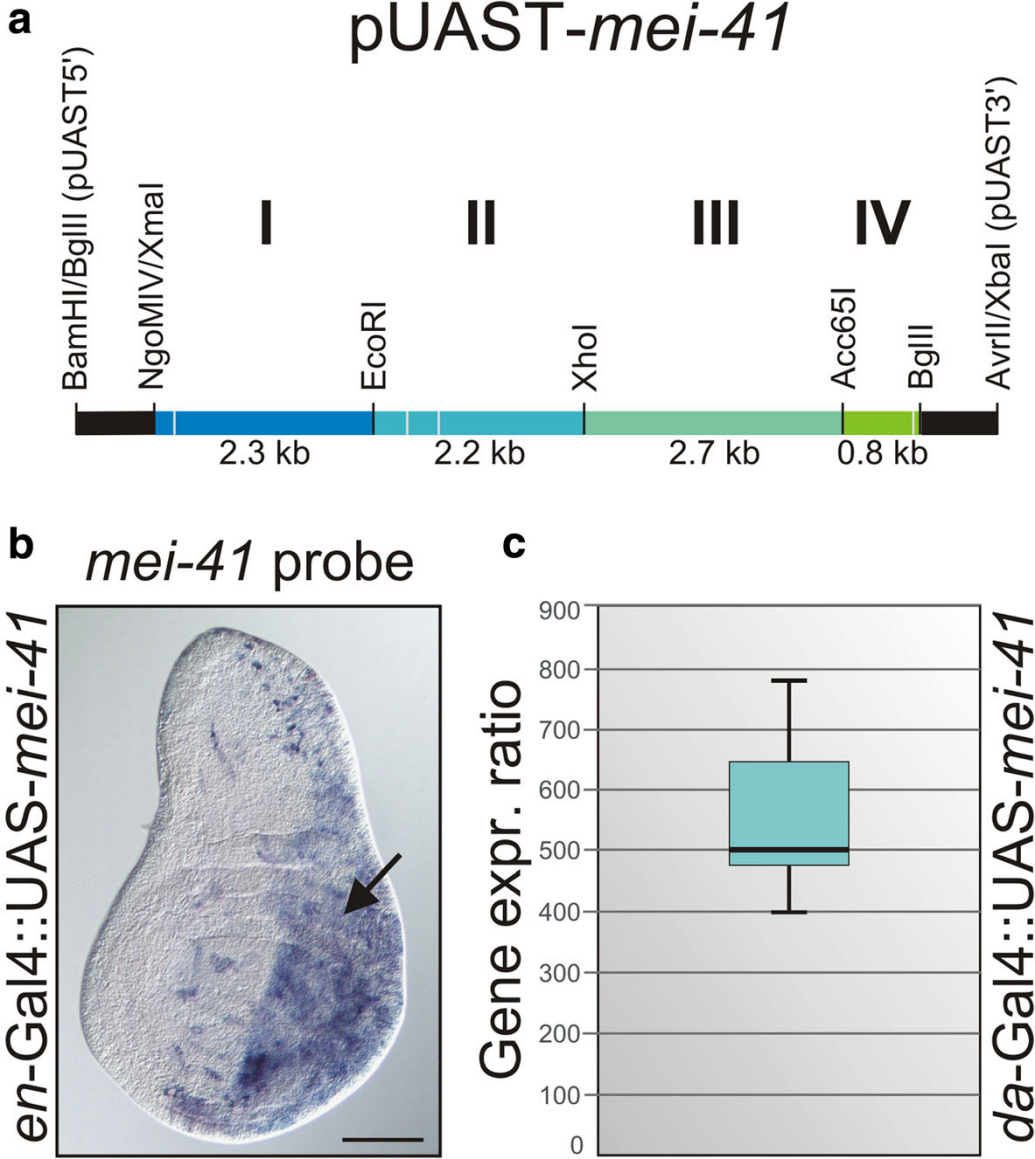
Generation of a UAS-mei-41 overexpression line. (**a**) Cloning scheme of the pUAST-*mei-41* construct. Coloured line corresponds to the *mei-41* gene; intron positions are depicted as pale dashes. Segments derived from plasmid vectors are depicted in black and are not to scale. Genomic *mei-41* DNA was PCR-amplified in fragments I-IV**,** which were eventually merged and shuttled into pUAST transformation vector in the correct 5′-3′ orientation. (**b**) UAS-*mei-41* flies were crossed to *en*-Gal4-GFP, and induction of *mei-41* expression was visualized in the posterior compartment of a wing imaginal disc by in situ hybridization (arrow). Size bar represents 100 μm. (**c**) Overexpression of *mei-41* was quantified by qRT-PCR. To this end, UAS-*mei-41* was ubiquitously induced with *da*-Gal4, and mRNA isolated from third instar larvae. Compared to control (*da*::*lacZ*), *mei-41* is about 500-fold overexpressed. Data were assembled from three biological and two technical replicates. Mini-max depicts 95% confidence, median corresponds to expression ratio. *Gapdh* and *beta*-*Tubulin56D* served as reference genes. Efficiencies for *mei-41* (0.92), for *gapdh* (0.94) and for *beta*-*Tubulin56D* (0.95) were accounted for determining relative quantities [44]

Aiming for a deeper understanding of the *Drosophila* DNA damage response, we concentrated on *meiotic 41* (*mei-41)*, the *Drosophila* checkpoint kinase ATR homologue and key player of this process [24, 25]. To study the consequences of *mei-41* overexpression, we generated a pUAST-*mei-41* construct allowing for tissue-specific induction during fly development with the help of the Gal4/UAS-system [34]. The *mei-41* locus contains four small introns (59 bp, 57 bp, 74 bp, 64 bp) and covers more than 8 kb; there is no complete cDNA available (7899 bp) (see http://flybase.org for further details). We therefore decided to PCR-amplify genomic DNA in four smaller fragments to be fused to cover the entire open reading frame, and clone it into the pUAST vector (Fig. 1a). Transgenic lines were established, tested and a third chromosomal insertion (3.3) line was used for further experiments. Overexpression of *mei-41* in the posterior compartment of wing imaginal discs using *en*-Gal4-GFP was demonstrated by in situ hybridization with a *mei-41* specific probe (Fig. 1b). Moreover, qRT-PCR revealed about a 500-fold induction when UAS-*mei41* was ubiquitously induced with *da*-Gal4 during larval development relative to the endogenous *mei-41* expression levels (Fig. 1c). We conclude that the UAS-*mei-41* construct is well suited for overexpression studies during *Drosophila* development.

### Overexpression of mei-41 affects entry into mitosis

**Fig. 2:**
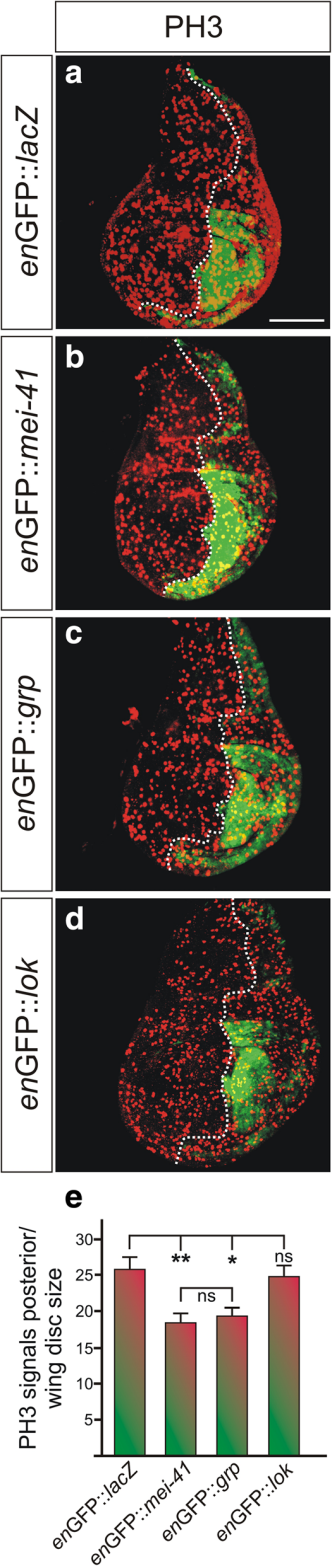
Overexpression of mei-41 induces G2/M cell cycle arrest. (**a**-**d**) The checkpoint kinases *mei-41, grp* and *lok* (*chk2*) as indicated were overexpressed in the posterior compartment of wing imaginal discs using *en*-Gal4-GFP (green) (b-d). UAS-*lacZ* served as control (a). To image M phase, discs were stained with anti-Phospho-Histone H3 (PH3) antibodies (red) indicating the number of mitotic cells. GFP labelling was used to determine the antero-posterior boundary (dotted line). (**e**) Quantification of PH3 signals showed significant downregulation upon overexpression of *mei-41* and *grp* but not of *lok*. Significance was tested by ANOVA two-tailed Tukey-Kramer approach (** *p* < 0.01; * *p* < 0.05; ns: not significant). Bars represent standard error (SEM) from 13 to 17 analyzed discs per genotype. Size bar represents 100 μm in all panels

Primary to DDR is checkpoint activation, i.e. slowing down the entry into mitosis to allow time for repair [5]. We wondered whether the overexpression of *mei-41* without further activation by DNA damaging compounds might suffice to affect cell cycle regulation. To this end, we induced *mei-41* in the posterior compartment of the wing disc using *en*-Gal4-GFP, and monitored cells in M phase of the cell cycle (Fig. 2). Cells in mitotic phase were visualized with anti-Phospho-Histone H3 (PH3) antibody staining and counted in the posterior compartment. Then their numbers were related to the whole wing disc size. As negative control, we overexpressed UAS-*lacZ*. Moreover, we included both downstream kinase Chk1/2 homologues *grapes (grp)* and *loki (lok)*, since overexpression studies so far had not included cell cycle analyses in imaginal tissue [10, 16, 18]. It has been shown earlier that *grp* has a major role in the DNA replication checkpoint, whereas the primary role of *lok* is p53-mediated apoptosis in response to IR [16, 18, 19, 25, 28, 35].

Quantification of the results allowed us to uncover a significant reduction in the number of mitotic cells upon the overexpression of either *mei-41* or *grp,* but not of *lok* (Fig. 2a-e). This is in agreement with earlier reports that *mei-41* and *grp* are involved in cell cycle arrest in *Drosophila* cells and tissues, with a minor contribution of *lok* [16, 18, 25, 28]. We confirmed that the reduced number of mitotic cells after *mei-41* or *grp* overexpression were not a consequence of apoptosis, as no increase of cleaved Caspase-3 activity was detected in the posterior compartment of the discs (see Additional file 1: Figure S1). Our results therefore demonstrate that both, *mei-41* and *grp,* act on cell cycle regulation in a dose dependent manner, in contrast to *lok.* Our data conform to the requirements of *mei-41* and *grp* in G2/M checkpoint function [24, 25]. Moreover, they demonstrate that our newly generated inducible *mei-41* construct is a valuable tool for further analysis of *mei-41* roles in a gain-of-function background, complementing the information gathered so far in a *mei-41* loss-of-function background.

### Ectopic induction of mei-41 during imaginal development reduces adult tissue size

**Fig. 3:**
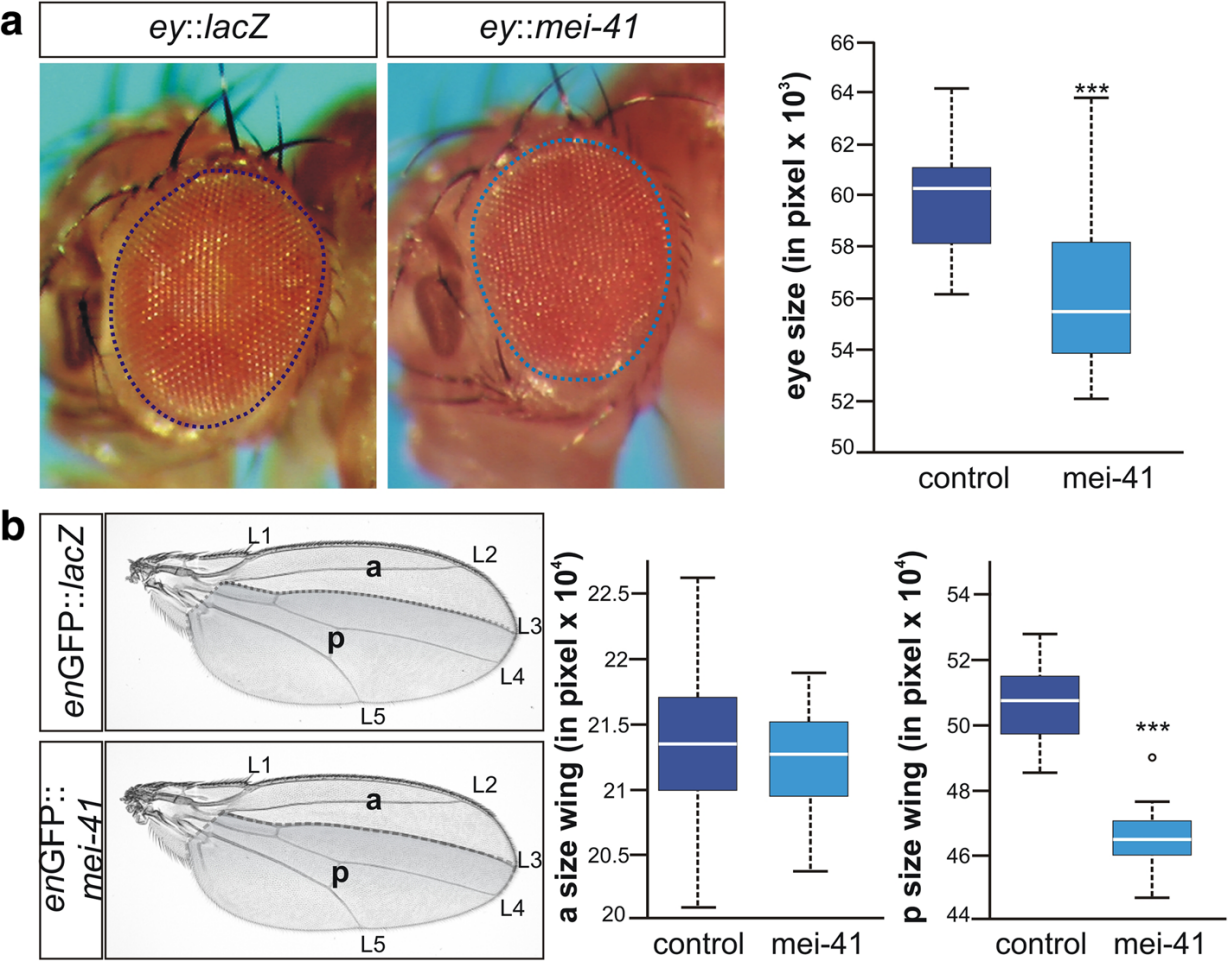
Overexpression of mei-41 results in smaller adult tissue size. (**a**) UAS-*mei-41* was overexpressed during eye development using *ey*-Gal4; likewise was UAS-lacZ serving as control. Eye size of adult female flies was measured as indicated. Compared to the control, *mei-41* overexpression caused a significant decrease in eye size (*n* ≥ 26). (**b**) Effects on tissue size of the overexpression of UAS-*mei-41* with *en*-Gal4-GFP in the posterior compartment of the developing wing were compared to those of the control UAS-*lacZ* (*n* ≥ 20). Size of anterior and posterior compartments was measured as indicated. Whereas no difference was detected in the anterior (a), the posterior compartment (p, shaded grey) was significantly smaller when *mei-41* was overexpressed. Longitudinal veins L1-L5 are labelled. Data are depicted as boxplots with center lines showing the medians, box limits indicate the 25th and 75th percentiles as determined by BoxPlotR software; whiskers extend 1.5 times the interquartile range. Statistics were done using ANOVA, and significance determined with Dunnett’s approach (*** *p* < 0.001)

To study the effects of *mei-41* overexpression on adult tissue size, we ectopically induced *mei-41* during proliferative phases of eye and wing development. Overexpression of *mei-41* in the anterior part of the developing eye disc using *ey*-Gal4 resulted in a profound reduction of adult eye size (Fig. 3a). This growth defect is not restricted to the eye since overexpression of *mei-41* in the posterior compartment of the developing wing disc using *en*-Gal4-GFP reduced its size within the adult wing significantly, whereas the size of the anterior compartment was unaffected (Fig. 3b). We did not observe any disturbance of wing and venation morphology, implying that growth but not differentiation or patterning of the tissue was affected (Fig. 3b).

So far, our data suggest that overexpression of *mei-41* is sufficient for the induction of a cell cycle arrest without affecting development. The effect is very mild though, and only apparent in a quantitative approach, which may be explained by the lack of kinase activation by genotoxic stress. We cannot rule out the formal explanation, however, that the excessive amounts of Mei-41 kinase may generally interfere with other aspects of cell growth and proliferation. For example, ATM/ATR kinases must be tightly regulated in order to prevent aberrant activation of DDR. It is thought that the availability of specific protein cofactors, required for kinase recruitment to DNA damage sites, restricts kinase activity. ATR forms a heterodimer with its obligate partner ATRIP during the sensing of DNA damage in the course of DDR (reviewed in [6, 8]), and so does *Drosophila* Mei-41 with the ATRIP homologue Mus 304 [25]. We propose that Mus 304 may be limiting the effects of *mei-41* overexpression, as the gene is expressed only about threefold of *mei-41* (http://flybase.org).

### Irradiation induced G2/M checkpoint is extended in mei-41 overexpressing cells

**Fig. 4:**
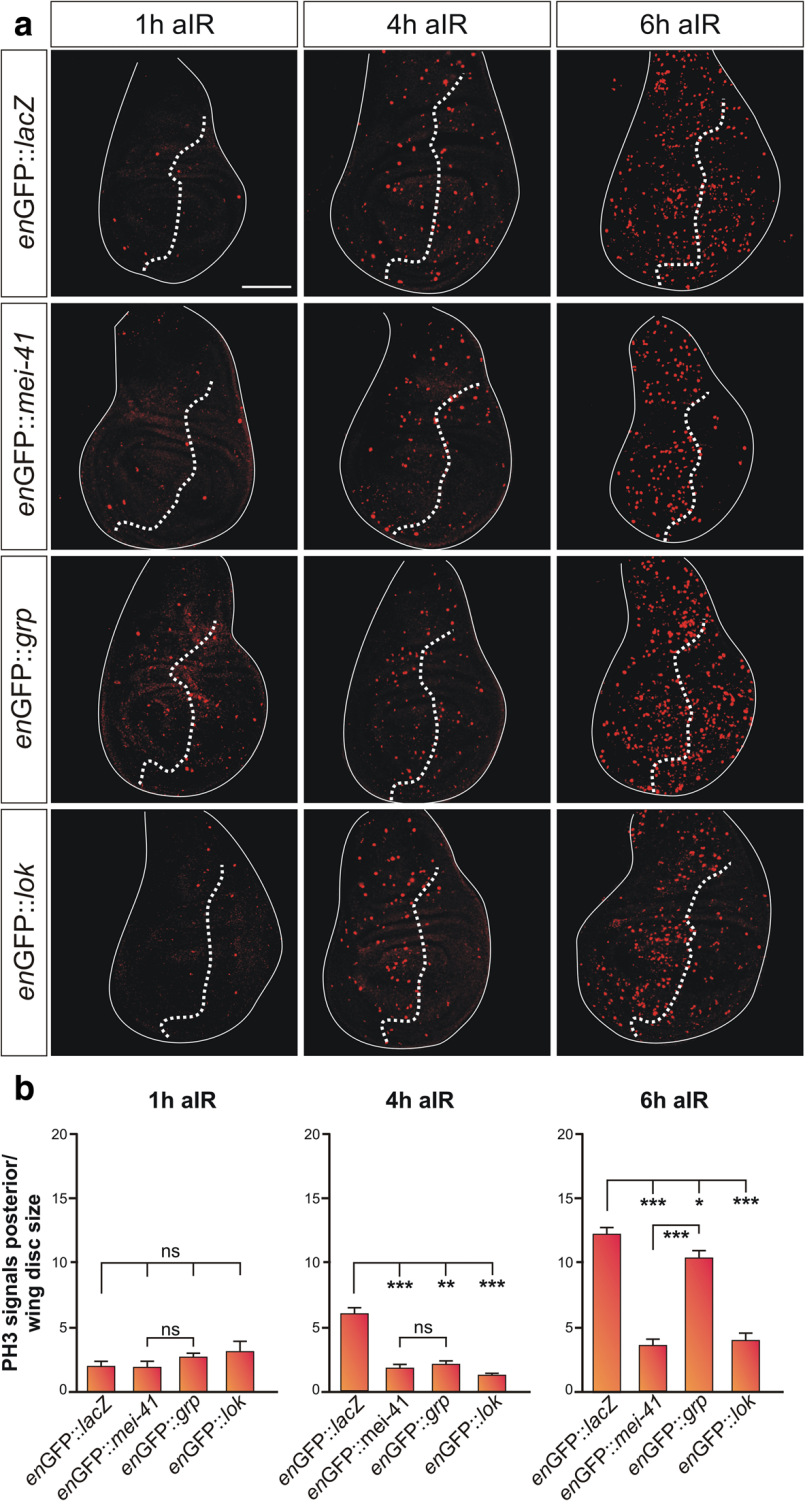
Cell cycle re-entry is delayed in cells overexpressing mei-41. (**a**) The indicated kinase or the lacZ control was overexpressed in the posterior compartment of wing imaginal discs using *en*-Gal4-GFP. Third instar larvae of the given genotypes were irradiated with 40 Gy and allowed to recover for one, four and six hours (1 h, 4 h, 6 h aIR) respectively, before dissection of the wing imaginal discs. Mitotic cells are highlighted by Phospho-Histone H3 antibody staining (red). GFP labelling was used to determine the antero-posterior boundary (indicated by the dotted line). IR-stress caused a cell cycle arrest, only few cells appear in M phase one hour after irradiation. Entry into mitosis reappears with time in the control (*en*GFP::*lacZ*), as well as in the anterior compartment of all genotypes as DNA damage response proceeds. Not so in the posterior compartment, though, where either *mei-41* or *lok* is overexpressed. However, overexpression of *grp* allowed the cell cycle to resume 6 h post-IR. Size bar represents 100 μm in all panels. (**b**) Quantification of PH3 signals in the posterior compartment reveals no significant difference amongst the different genotypes 1 h after irradiation (aIR). Re-entry into mitosis is observed in the wild type but not the other genotypes at 4 h aIR. Even at 6 h aIR, significantly less mitotic cells are observed in the posterior compartments overexpressing either *mei-41* or *lok*, whereas *grp* overexpression had only minor lasting effects. *** *p* < 0.001; ** *p* < 0.01; * *p* < 0.5; ns, not significant) according to ANOVA two-tailed Tukey-Kramer approach for multiple comparisons. Bars represent standard of the mean (SEM). At least 16 wing discs were analyzed for each genotype and time point

We know from the literature that *mei-41* is indispensable for the irradiation induced G2/M checkpoint [13, 22, 25]. Likewise, the combined activity of *grp* and *lok* is required*,* with a minor contribution of the latter [16, 18, 25, 28]. Accordingly, we wondered how overexpression of *mei-41, grp* or *lok* may affect the cellular response to irradiation. Larvae overexpressing the respective constructs in the posterior compartment of imaginal discs were exposed to 40 Gy IR. PH3 staining uncovered cells in early mitosis in wing imaginal discs: as expected, IR-induced a G2/M checkpoint as very few cells entered mitosis 1 h after irradiation (Fig. 4a, b). Cell cycle arrest is induced and maintained to allow sufficient time for DNA repair, and eventually the cell to recover from DNA damage and resume cell cycle. Accordingly, cells start to re-enter mitosis, seen by increasing numbers of PH3 positive cells over time. In the control, we observed rising numbers of PH3 positive cells already at 4 h post-IR, becoming considerably numerous 6 h later (Fig. 4a, b). A likewise increase was observed in the anterior compartment of the irradiated discs from larvae overexpressing either *mei-41, grp* or *lok*. In the posterior compartment, however, i.e. in cells overexpressing *mei-41*, re-entry into the cell cycle was considerably hampered even after 6 h recovery time (Fig. 4a, b), presumably by keeping the DSB damage checkpoint active. We expect both, the endogenous as well as the overexpressed Mei-41 kinase to be activated in response to IR-stress. Consequently, cells are expected to be flooded by activated Mei-41, keeping them from resuming mitosis entry. Even several hours post-IR, activated Mei-41 levels apparently surpass the threshold for a G2/M checkpoint.

Overexpression of *grp* had a much milder effect: 4 h after irradiation, cell cycle had not yet resumed, whereas after 6 h, the number of mitotic cells was approaching that of the control (Fig. 4a, b). These data indicate that *grp* overexpression slowed down cell cycle re-entry, but not as efficiently as *mei-41* overexpression. This result may be expected, since *grp* encodes the effector kinase downstream of *mei-41*: IR-stress activates endogenous Mei-41 kinase which phosphorylates and activates the effector kinase Grp. In the presence of abundant Grp protein, this response will be intensified and longer lasting. Therefore, we expect cell recovery to lag behind, i.e. *grp* to mirror but not to match *mei-41* overexpression.

Overexpression of *lok*, however, had a similar effect as *mei-41* overexpression (Fig. 4a, b), which, at first glance, came as a surprise: cell cycle re-entry appeared delayed even at 6 h after irradiation, i.e. the number of PH3 positive cells was still reduced. However, we had not observed any effect of *lok* overexpression on cell cycle regulation in the absence of IR-stress (Fig. 2a), in agreement with the many reports on the supplemental role of *lok* for the G2/M checkpoint [25, 28]. Instead, the specific role for *lok* in the activation of p53-mediated cell death is well established [15, 16, 25]. Thus, we wondered whether the lack of PH3 positive *lok* overexpressing cells several hours post-IR indeed reflected failure of cell cycle re-entry, or alternatively might be explained by IR-induced *lok*-mediated apoptosis. To this end, although no signs of cell death were observed in unstressed discs where *lok* was overexpressed (Fig. 2), we repeated the experiment, now addressing apoptosis induction by IR-stress with 6 h recovery time. Indeed, in response to IR-stress accumulation of cleaved Caspase-3 was detected most prominently in the central part of the wing disc in all genotypes (see Additional file 1: Figure S2). In contrast to the discs overexpressing either *lacZ*, *mei-41* or *grp,* however, *lok* overexpressing cells showed a much stronger apoptotic response than any of the others (see Additional file 1: Figure S2). We conclude that the lower numbers of PH3 positive cells present in *lok* overexpressing tissue may result primarily from an increase in apoptosis rather than from a delay of cell cycle re-entry.

### Mobility shift of Grp after mei-41 overexpression

**Fig. 5:**
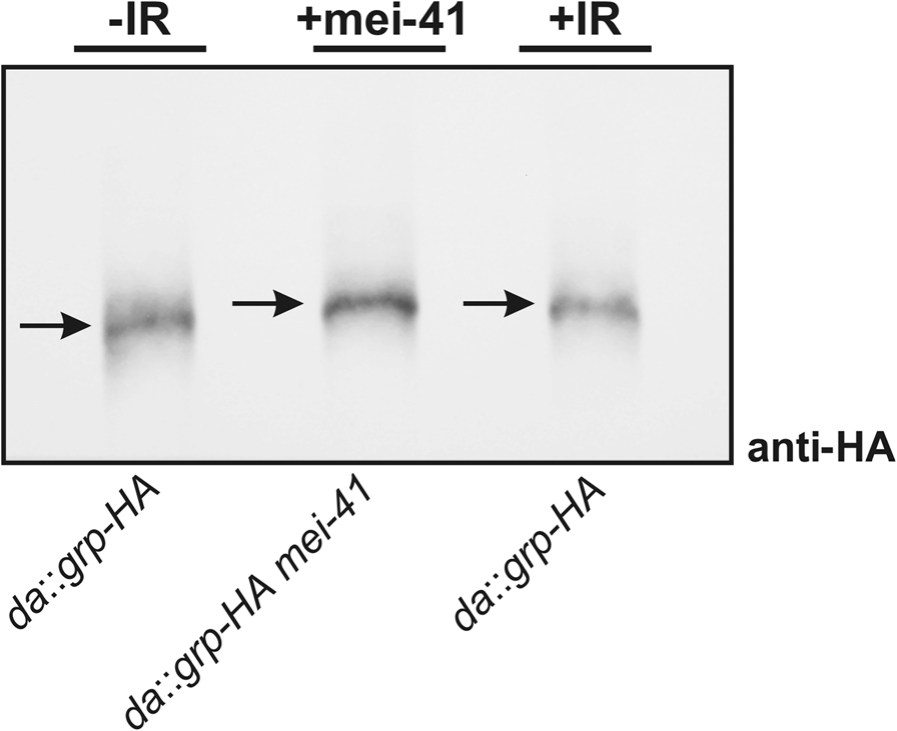
Overexpression of mei-41 results in an electrophoretic mobility shift of Grp. Mobility of HA-tagged Grp protein was analyzed in response to either irradiation (1 h after 40 Gy IR) or the overexpression of *mei-41*. Western analysis combined with Phos-Tag™-PAGE was performed using anti-HA antibodies and proteins extracts from imaginal discs of 25 larvae from each genotype. Note similar mobility shift in response to IR or *mei-41* overexpression indicative of a phosphorylation of Grp protein. (Genotypes are: *da*-Gal4/+; *da*-Gal4/UAS-*grp-HA* and *da*-Gal4/+; *da*-Gal4/UAS-*grp-HA* UAS-*mei-41*)

Using *Drosophila* Schneider S2 cells, it has been demonstrated that Grp is phosphorylated in response to DNA damage or incomplete DNA replication, and that this phosphorylation was dependent on the presence of Mei-41 [28]. Presumably, the *Drosophila* ATR homologue Mei-41 phosphorylates Grp in the process of DDR, comparable to what is known from other organisms like yeast, *Xenopus* and also mammals [2, 3]. As we had seen a dose dependency for *mei-41* on checkpoint induction (Fig. 2), we wondered, whether the sole overexpression of *mei-41* may suffice to induce Grp phosphorylation, i.e. induce a bona fide DNA damage response. To this end, *mei-41* was ubiquitously overexpressed together with a HA-tagged form of Grp using *da*-Gal4. Larval protein extracts from imaginal discs were separated by Phos-Tag™ PAGE to increase the separation of phosphorylated from unphosphorylated proteins, followed by Western blotting. For negative control, larvae just overexpressing HA-tagged Grp were used; for positive control such larvae had been subjected to 40 Gy IR-stress. Indeed, overexpression of *mei-41* resulted HA-tagged Grp to migrate more slowly compared to the negative control, but similar to the one detected 1 h post-IR (Fig. 5). These results suggest, that overexpression of *mei-41* is sufficient to induce DDR at low levels, i.e. to activate, respectively phosphorylate its downstream target Grp in vivo.

### Activation of the p53R-GFP biosensor can be achieved by overexpression of mei-41

**Fig. 6:**
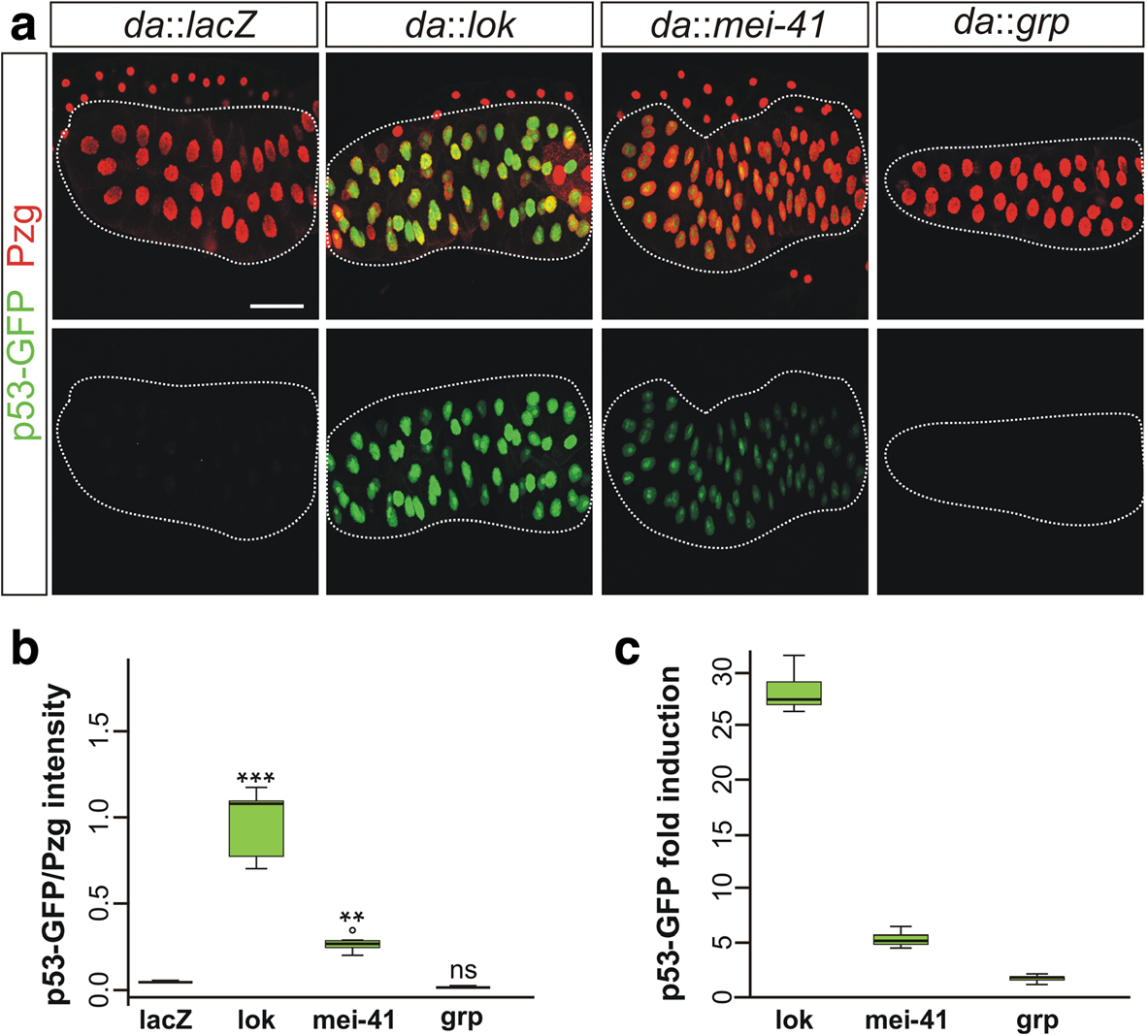
Response of the p53R-GFP reporter on mei-41 overexpression. (**a**) In response to p53 activation, nuclear GFP is expressed from the p53R-GFP reporter [36]. This system was used to assay p53 activation in consequence of the overexpression of either *lok*, *mei-41* or *grp*; *lacZ* served as control. Salivary glands were analyzed; their nuclei visualized with the nuclear marker Pzg [39]. In contrast to *lacZ* and *grp*, overexpression of *lok* and to a lesser degree *mei-41* resulted in a robust induction of the p53R-GFP reporter. Size bar represents 100 μm in all panels. (**b**) Quantification of nuclear p53R-GFP intensity was determined relative to the mean intensity of the nuclear marker protein Pzg (*n* = 84). Induction of *lok* strongly induced p53R-GFP nuclear accumulation. Also *mei-41* caused a significant nuclear accumulation of p53R-GFP, whereas *grp* did not. *** *p* < 0.001; ** *p* < 0.01; ns. not significant according to ANOVA two tailed Dunnet’s approach. (**c**) Expression levels of GFP were quantified by qRT-PCR in third instar larvae. Overexpression of *lok* and *mei-41* with *da*-Gal4 considerably increased p53R-GFP reporter gene activity in relation to the lacZ-control: *lok* 27.5-fold, *mei-41* 4.9-fold and *grp* 1.6-fold. Data were assembled from four biological and two technical replicates. Mini-max depicts 95% confidence, median corresponds to expression ratio. As reference genes, *cyp33* and *Tbp* were used. Efficiencies for *GFP* (0.91), for *cyp33* (0.96) and *Tbp* (0.95) were accounted for in determining relative quantities [44]

As outlined above, *mei-41* mutants display checkpoint defects that are only matched by *grp; lok* double mutants, implying that Mei-41 may not only act on Grp but also, to a lesser degree, on Lok [16, 25, 28]. Genetic and molecular data imply that Lok, but not Grp, is an activator of p53 in response to DNA damage in *Drosophila* [16, 25]. To investigate a potential cross-regulatory effect of the *Drosophila* ATR homologue Mei-41 on p53 activity we made use of a p53R-GFP biosensor, where p53 activation is reflected by nuclear GFP accumulation [36]. This p53R-GFP biosensor is activated during meiosis in the female germline, as well as in response to genotoxic stress in somatic tissues [36–38]. Thus, it is well suited to determine whether *mei-41* overexpression alone may suffice to effect p53 activation. UAS-*mei-41* was ubiquitously overexpressed with *da*-Gal4 in the background of the p53R-GFP reporter, and the giant salivary gland nuclei were examined for accumulation of GFP. GFP signal intensity was measured, using nuclear Putzig (Pzg) protein [39] as internal standard. For control, p53R-GFP nuclear localization was evaluated after ectopic induction of either Lok or Grp as well. As expected from its ability to activate p53 [16, 18, 19], overexpression of *lok* caused a strong nuclear GFP signal, which was well above *lacZ* control, demonstrating the reliability of our test system (Fig. 6a, b). Interestingly, overexpression of *mei-41* was sufficient to induce nuclear p53R-GFP accumulation, albeit much weaker than that of *lok* (Fig. 6a, b). In contrast, Grp was unable to trigger measurable GFP signals (Fig. 6a, b). In addition to the visual assessment, qRT-PCR measurements of GFP expression levels were conducted on larval imaginal discs. They uncovered a 27-fold and 5-fold increase of p53-GFP reporter expression, respectively, in response to ubiquitous *lok* and *mei-41* overexpression in comparison to the *lacZ* control (Fig. 6c). These data provide unambiguous evidence that ectopic Mei-41 is able to induce p53 activity. Whether this activation is direct, as shown for ATM as well as ATR (reviewed in [6, 8]), or indirect via Lok, requires further investigations. Our newly established UAS-*mei-41* construct is well suited to facilitate future analyses.

## Discussion

In this work, we show that *mei-41* and *grp* act on cell cycle regulation in a dose dependent manner*,* conforming to the requirements of *mei-41* and *grp* in G2/M checkpoint function [24, 25]. Whereas developmental patterning is unaffected, a cell cycle arrest and proliferation defects result from the overexpression of *mei-41*. Moreover, we unambiguously show that p53 activity is induced by ectopic Mei-41. We do not know, however, whether this activation is direct, as shown for ATM as well as ATR (reviewed in [6, 8]), or indirect via Lok. The defects resulting from Mei-41 overexpression are very mild, however, both regarding cell cycle delay, cell proliferation or apoptosis, compared to a normal irradiation response. There are several explanations. For example, during genotoxic stress Mei-41 kinase is activated by phosphorylation, which does not follow the overexpression. Moreover, Mei-41 heterodimerizes with its obligate partner Mus 304 [25] during damage response [6, 8]. In our overexpression experiments, Mus 304 may be limiting, allowing only for the mild effects observed. Finally, excessive amounts of Mei-41 kinase resulting from the overexpression may generally interfere with other aspects of cell growth and proliferation.

## Conclusions

Overexpression of the *Drosophila* ATR homologue *mei-41* during imaginal development is sufficient to initiate a cellular response resembling the DNA damage response, which is reflected by the induction of a G2/M checkpoint, growth retardation, apparent phosphorylation of the effector kinase Grp (*Drosophila* Chk1), as well as the activation of p53. The rather subtle effects presumably result from a lack of Mei-41 kinase activation by genotoxic stress, as IR resulted in long-lasting checkpoint activation. Moreover, the observation of p53 activation in response to *mei-41* overexpression indicates cross-talk of ATR/ATM pathways also in *Drosophila* that may involve the effector kinase Lok (*Drosophila* Chk2).

## Methods

### Cloning of pUAST-mei-41 and generation of transgenic flies

Four fragments covering *mei-41* were PCR-amplified from genomic DNA, and subcloned individually in pBT vector (Stratagene, La Jolla, USA) (fragments I-III) or pGX-attP [40] (fragment IV) to obtain the following subclones: pBT 2.3 kb *Ngo*MIV/*Xma*I - *Eco*RI fragment I (translation start to *Eco*RI); pBT 2.2 kb *Eco*RI-*Xho*I fragment II; pBT 2.7 kb *Xho*I-*Acc*65I fragment III; pGX-attP 0.8 kb *Acc*65I - *Bgl*II fragment IV (translation stop). Subsequently fragments I-III were fused by successive cloning in pBT vector. The insert was excised as *Sac*II/*Acc*65I fragment and cloned into likewise opened pGX-attP harbouring fragment IV. The full length genomic *mei-41* construct was excised with *Bam*HI/*Avr*II and subsequently shuttled into *Bgl*II/*Xba*I of pUAST vector [34] and sequence verified. Three independent transgenic fly lines were established by P-element mediated germline transformation [41]. All three lines were functionally tested for ectopic expression of *mei-41* RNA by in situ hybridization of imaginal discs. The subsequent functional assays were performed with line UAS-*mei-41* (3.3) inserted on the third chromosome.

### RNA expression analyses: In situ hybridization and qRT-PCR

In situ hybridization on larval wing discs was performed with digoxigenin-labelled DNA probes of *mei-41* according to standard protocols [42]. Quantitative RT-PCR was performed as outlined earlier [38, 43]. With the *PolyATract® System 1000 kit* (Promega Mannheim, Germany) poly(A)^+^ RNA was isolated from 20 total third instar larvae for the quantification of UAS-*mei-41* expression, and from imaginal discs only attached to the mouth hook from 20 third instar larvae for p53R-GFP biosensor quantification. Real time qPCR was conducted with *Blue S’Green qPCR kit* (Biozym, Hessisch-Oldendorf, Germany) on 6 ng of cDNA in 10 μl end volume using MIC magnetic induction cycler (*bms*, Pots Point, Australia) including target and no-template controls. Absence of genomic DNA was tested in a non-RT control. As internal references for *mei-41* expression, *βTub56D* and *gapdh2* were used*,* whereas *cyp33* and *Tbp* served as internal references in the case of *p53R*-GFP biosensor quantification. The references were selected based on variance and Cq values. Relative quantification of the data was performed with *micPCR* software Version 2.6.0 based on *REST* taking target efficiency into account [44]. At least three biological and two technical replicates were performed. The following primer pairs were used (5′ - > 3′):

- *mei-41* upper, CTC CTG CAA GAC TTT AAT TCG CTC AC
  - lower, GCG TTG GCT GCA TGT ACT TCT CA
- *βTub56D* PP17563 DRSC FlyPrimer bank [45]
- *gapdh*2 PP2976 DRSC FlyPrimer bank [45]
- *GFP* upper, TCAAGGACGACGGCAACTACAAGAC
  - lower, TCACCTTGATGCCGTTCTTCTGC
- *cyp33* PP14577 DRSC FlyPrimer bank [45]
- *Tbp* PP1556 DRSC FlyPrimer bank [45]

### Fly work and immunochemistry

Flies were raised on standard corn-molasses food at 25 °C. The following strains were used: *da*-Gal4 (BL55849); *en*-Gal4-GFP [46], UAS-*lacZ* (BL8530), UAS-*mei-41* (this work), UAS-*grapes.ORF.3xHA* (F000934; obtained from FlyORF; Zürich, Switzerland) [47], UAS-*chk2* (*lok*) (gift of U. Abdu) [48], UASp-*lacZ* [49], *p53R*-GFP [36]*.* Staining of third instar wing imaginal discs or salivary glands was done according to standard protocols as described earlier [38, 50] using the following antibodies: rabbit anti-GFP (1:100; Santa Cruz Biotech, Dallas, USA), guinea pig anti-Pzg (1:1000) [39], rabbit anti-cleaved Caspase-3 (1:250; Cell Signaling, Germany) and rabbit anti-Phospho-Histone H3 (PH3) (1:50; Cell Signaling, Germany). Secondary antibodies from goat or donkey, coupled to FITC or Cy3, were obtained from Jackson Immuno-Research Laboratories (Dianova, Hamburg, Germany). Larval tissue was documented by confocal microscopy using a MRC1024 confocal scan head coupled to a Zeiss Axiophot (Carl Zeiss AG, Oberkochen, Germany) and LaserSharp 2000 imaging software. Pictures were compiled with Corel Photo Paint and Corel Draw software.

### Ionizing radiation (IR) treatment and Phos-Tag™ based mobility shift detection

Third instar larvae were irradiated with 40 Gy using Elektra Versa HD linear accelerator (Elektra Instrument AB; Stockholm; Sweden) at the Marienhospital Stuttgart. To investigate phosphorylation of Grp protein, 25 third instar larvae of the genotype *da*-Gal4/+; *da*-Gal4/UAS-*grp-HA* were irradiated with 40 Gy. After 1 h recovery time, imaginal discs connected to the mouth hook were isolated and homogenized in 50 μl binding buffer (20 mM HEPES pH 7.6, 150 mM KCl, 2.5 mM MgCl_2_, 10% glycerol, 0.05% NP-40, 1 mM DTT, ROCHE complete ULTRA protease inhibitor mini tablet). The unirradiated controls as well as the *da*-Gal4/+; *da*-Gal4/UAS-*grp-HA* UAS-*mei-41* larval tissues were treated likewise. The homogenates were separated in 10% SDS-PAGE including 50 μM Phos-Tag™ Acrylamide solution (#AAL-7, Wako Chemicals GmbH, Neuss, Germany) and 100 μM MnCl_2_ at 70 V for 22-24 h at 8 °C. After blotting on PVDF membrane (BioRad, Munic, Germany), HA-tagged Grapes was detected with rat anti-HA (1:2500, Roche Diagnostic, Basel, Switzerland), and secondary anti-rat antibody coupled to alkaline phosphatase (1:1000; Jackson Immuno-Research Laboratories via Dianova, Hamburg, Germany).

### Documentation and statistical evaluation of larval and adult tissue

Cells in M phase within the posterior compartment were counted based on PH3 signals. The posterior compartment was determined by GFP labelling from *en*-Gal4-GFP [46]. Cell number was related to total size of the respective wing discs using *ImageJ*. p53R-GFP expression in salivary glands was examined by measuring signal intensity of 12 nuclei from seven different glands each (*n* = 84 nuclei), using the mean intensity of Pzg signals as internal standard. Wings from female flies were dehydrated in ethanol, mounted in Euparal (Roth, Karlsruhe, Germany) and documented with an ES120 camera (Optronics, Goleta CA, USA) connected to a Zeiss Axiophot (Carl Zeiss AG, Jena, Germany) using Pixera Viewfinder software, version 2.0. Female flies were etherized before taking pictures from the heads with an ES120 camera coupled to a Leica Wild M3C Stereomicroscope (Leica, Wetzlar, Germany). Size of female eyes (UASp-*lacZ*/+; *ey*-Gal4/+ and *ey*-Gal4/+; UAS-*mei-41*/+) or wings (UASp-*lacZ*/+; *en*-Gal4-GFP/+ and *en*-Gal4-GFP/+; UAS-*mei-41*/+) was measured using *Image J*. Statistical analysis was conducted by ANOVA using a two-tailed Tukey-Kramer or Dunnett’s test for multiple comparisons. ***p < 0.001 highly significant; **p < 0.01 very significant; *p < 0.05 significant; not significant (ns) *p* > 0.05. Box plots were compiled using the online plotting tool BoxPlotR (http://shiny.chemgrid.org/boxplotr/).

## Additional file

Additional file 1: Analysis of apoptosis. (PDF 9148 kb)

## Abbreviations

ATM: Ataxia-Teleangiectasia Mutated
ATR: ATM and Rad3-related
Chk1, Chk2: Checkpoint kinase 1, 2
*da*: *Daughterless*
DDR: DNA damage response
DSB: DNA double strand breaks
*en*: *Engrailed*
GFP: Green fluorescent protein
*grp*: *Grapes*
IR: Ionizing radiation
*lok*: *Loki*
*mei-41*: *Meiotic 41*
PH3: Phospho-Histone H3
