# Supplementary material for: Overexpression of the Drosophila ATR homologous checkpoint kinase Mei-41 induces a G2/M checkpoint in Drosophila imaginal tissue
Source: Hereditas. 2018 Sep 6;155:27. doi: 10.1186/s41065-018-0066-4 (PMC6125995; doi:10.1186/s41065-018-0066-4)
Supplement: Supplementary file 1 — Analysis of apoptosis. (PDF 9148 kb) [file 41065_2018_66_MOESM1_ESM.pdf]

Additional File

to

Overexpression of the *Drosophila* ATR homologous checkpoint kinase Mei-41 induces a G2/M checkpoint in *Drosophila* imaginal tissue

Fabienne E. Bayer[1,¶], Mirjam Zimmermann[1,¶], Anette Preiss[1] and Anja C. Nagel[1]*

[1]Institut für Genetik, Universität Hohenheim, Garbenstr. 30, 70599 Stuttgart, Germany

[¶:] These authors contributed equally to this work.

*: corresponding author

e-mail: anja.nagel@uni-hohenheim.de

The Additional file contains the Figures S1 and S2

Figure S1 **Overexpression of *mei-41* has no effect on apoptosis**

Figure S2 **Increased apoptosis is observed in irradiated discs after *lok* overexpression**

Figure S1 Overexpression of *mei-41* has no effect on apoptosis

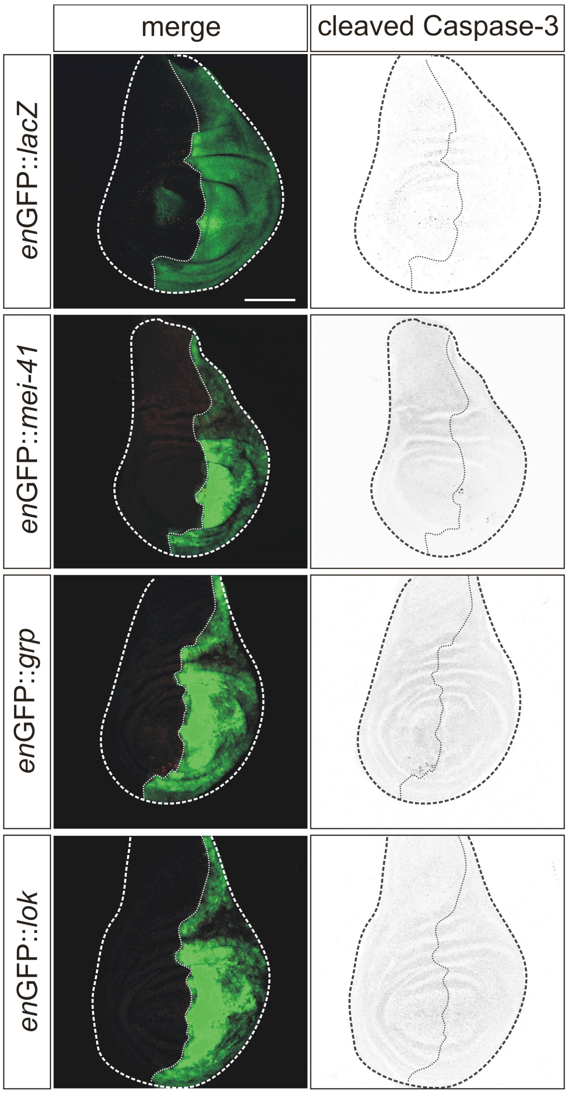

Figure S1

The checkpoint kinase genes *mei-41, grp,* and *lok* (Chk2) as indicated were overexpressed in the posterior compartment of wing imaginal discs using *en*-Gal4-GFP (shown in green in the merge panel). UAS-*lacZ* served as control. Cleaved Caspase-3 was detected by antibody staining (shown in red in the merge panel) to determine cell death induction in the given genotypes. The staining is shown in the right panel inversed for visibility purposes. Size bar represents 100 μm in all panels.

Figure S2 Increased apoptosis is observed in irradiated discs after *lok* overexpression

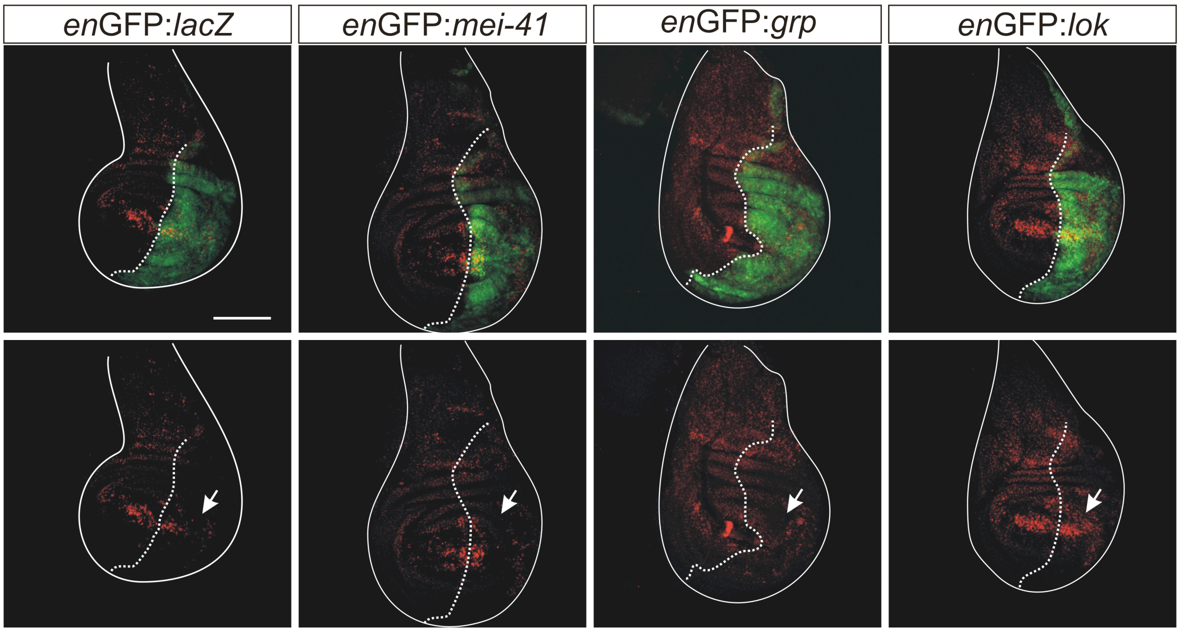

Figure S2

Either *lacZ, mei-41, grp* or *lok* was overexpressed with *en*-Gal4-GFP as indicated; larval imaginal discs were examined 6h after irradiation with 40 Gy. Cleaved Caspase-3 (red) was used to visualize the amount of cell death in consequence of IR-stress and recovery thereafter. The posterior compartment, highlighted with GFP (shown in green in the upper panel), is marked with a dotted line. Only the overexpression of *lok* but neither of *mei-41* nor *grp*, resulted in a pronounced enhancement of Caspase-3 activity (arrows), i.e. induction of apoptosis. Size bar represents 100 μm in all panels.
